# Effects of Particle Swarm Optimisation on a Hybrid Load Balancing Approach for Resource Optimisation in Internet of Things

**DOI:** 10.3390/s23042329

**Published:** 2023-02-20

**Authors:** Dorcas Dachollom Datiri, Maozhen Li

**Affiliations:** Department of Electronic and Electrical Engineering, Brunel University London, Uxbridge UB8 3PH, UK

**Keywords:** particle swarm optimisation, clustering, resource scheduling, resource allocation, resource optimisation

## Abstract

The internet of things, a collection of diversified distributed nodes, implies a varying choice of activities ranging from sleep monitoring and tracking of activities, to more complex activities such as data analytics and management. With an increase in scale comes even greater complexities, leading to significant challenges such as excess energy dissipation, which can lead to a decrease in IoT devices’ lifespan. Internet of things’ (IoT) multiple variable activities and ample data management greatly influence devices’ lifespan, making resource optimisation a necessity. Existing methods with respect to aspects of resource management and optimisation are limited in their concern of devices energy dissipation. This paper therefore proposes a decentralised approach, which contains an amalgamation of efficient clustering techniques, edge computing paradigms, and a hybrid algorithm, targeted at curbing resource optimisation problems and life span issues associated with IoT devices. The decentralised topology aimed at the resource optimisation of IoT places equal importance on resource allocation and resource scheduling, as opposed to existing methods, by incorporating aspects of the static (round robin), dynamic (resource-based), and clustering (particle swarm optimisation) algorithms, to provide a solid foundation for an optimised and secure IoT. The simulation constructs five test-case scenarios and uses performance indicators to evaluate the effects the proposed model has on resource optimisation in IoT. The simulation results indicate the superiority of the PSOR2B to the ant colony, the current centralised optimisation approach, LEACH, and C-LBCA.

## 1. Introduction

The internet of things (IoT), the umbrella word for extending the internet beyond smartphones and computers to a whole range of things such as appliances, smart sensors, cars, and traffic lights [1], requires effective management for supreme efficiency. Over the years, IoT’s increasing popularity and ubiquitous nature consisting of numerous distributed nodes with sensing, computing, and communication capabilities [2] implies an even greater increase in delayed- sensitive data generation that requires quick resources for execution [3]. The massive rise in data generation and consumption on account of the application’s requirement for higher data-rates, larger bandwidth, increased capacity, low latency, and high throughput [4] generates several challenges including but not limited to tremendous traffic pressure in the network and traditional cloud computing architectures [1]. The dynamically changing demand for resources with limited supply increases the risk of low quality of service (QoS) [5], consequently lowering users’ quality of experience (QoE). The optimisation of resource allocation techniques and satisfying the user QoS requirements are principal issues in an IoT-based cloud computing environment [6].

Real-world scenarios can all be described by the resource allocation problem (RAP); several formulations for the RAP have been proposed in accordance with different problem scenarios [7,8,9]. RAPs for IoT are large-scale with numerous facets; these RAPs in the IoT environment, referred to as multiple objective resource allocation problems (MORAP), are nondeterministic polynomial (NP)-complete and therefore cannot be handled by deterministic algorithms [7,8,10]. Over the years, NP algorithms such as evolutionary algorithms (EA), particularly genetic algorithms (GA), have been studied to find near-optimal solutions; however, GA algorithms have a tendency to reproduce a large number of infeasible solutions during search processes [10]. The limitations of GA prompted [7] therefore to propose a solution for the nonlinear MORAP, a particle swarm optimisation (PSO) metaheuristic clustering approach with focus on the Pareto-optimal solutions, that is, solutions that are not dominated by any other solutions, where no preference criterion is made better-off without making at least one preference criterion worse off. A Pareto optimal solution based on resource ready time is used to transform problems into multi-objective decisions to solve scheduling problems; it has been shown that the proposed Pareto optimisation algorithms produce optimal solutions [7,11]. Considering that IoT’s main function revolves around environment monitoring, data collection, and processing, prolonging the network’s lifespan by optimizing energy consumption has become a critical issue that necessitates utmost attention [12,13]. The inefficient utilisation of resources in IoT nodes may cause sensor and actuator nodes to be prematurely lost, due to their small battery power [13]. Consequently, edge computing (EC) paradigms, centring on edge nodes, have increasingly become popular for solving IoT’s MORAP; this shift in approach has brought about ample research on resource allocation (RA) as a means for managing traffic pressure and challenges associated with IoT and cloud computing architectures. It has been well established that the edge computing paradigm comes with some problems similar to cloud computing; such problems include scheduling service resources, ensuring quality of service (QoS), and combining different services [1,6,7].

Edge nodes entailing varying devices also comprise sensor nodes; these sensor nodes are more prone to sensing coverage, that is, the sensors capacity of supervising a specified area of interest; connectivity, specifically sensors’ communication capacity; and energy-consumption problems owing to its rechargeable battery constraints [12]. Wireless sensor networks (WSN), a subsection of IoT, can be considered a necessity for the development of IoT as they are incorporated in a wide range of applications such as smart-homes, smart-transport, and smart-healthcare [14]. WSN have repeatedly proven to be the bane of IoT problems as a malfunction in WSN’s performance will inevitably lead to some form of IoT system failure. Sensor edge nodes are plagued with low power and limited storage capacity; thus, when developing a model, it is necessary to consider energy dissipated due to activities. Prevalent approaches of the current state of the art are centralised algorithms with or without CH rotation properties that do not take into consideration the heterogeneous nature of IoT devices in terms of varying power, processing, and storage capacity. These existing systems assume all nodes perform equally; however, with this expectation comes sensitive latency issues and an inadequate load-balancing strategy alongside the inadequate optimisation of resources in IoT systems [7,10,14,15]. Load-balancing activities, a subset of the RAP, should most importantly decrease energy consumption whilst increasing QoS [5]. The work of [14] developed a centralised architecture-based clustering algorithm for load-balancing in IoT called C-LBCA. Using PSO for cluster formation, the developed C-LBCA algorithm advocates an architecture where the software-defined network (SDN) controller responsible for complex computations is implemented over the cloud with the aim of reducing functionality in the WSNs nodes. Presented extension simulation results in terms of network lifetime, energy dissipation, and volume of data sent to the sink validate the proposed C-LBCA. Although their work produced results indicating the significant extension of the battery life of IoT devices over the energy-aware clustering algorithm (PSO-C), stable election protocol (SEP), and low-energy adaptive clustering hierarchy (LEACH), their focus was limited to the effects of load balancing on the lifespan of WSN, and no outputs were presented with regard to the efficacy of the algorithm in overall system performance, especially when it is clear that the lifespan of the edge nodes including sensor nodes inadvertently affects the system’s turnaround time. Moreso, considering IoT’s increasing applicability to everyday living, their centralised network architecture approach is susceptible to multiple problems associated with nodal failure. Centralised topologies continuously expose IoT systems to heightened potential complexities and system failure with just a single point nodal failure.

In a bid to avoid the problem associated with potential node failure consequent of the centralised topology, the authors of [15] incorporated standards that allow for CH rotation; however, in this approach, the issue of inadequate maintenance persists and increases complexities. In their work, the authors of [15] developed a balanced energy-efficient (BEE) clustering algorithm that can elect CHs according to both energy consumption and sensor distributions extending the network’s longevity whilst maintaining the network’s coverage. Even though the authors of [15] generated results that indicated dominance over LEACH, hybrid energy-efficient distributed (HEED), and a non-cluster routing solution (direct routing), their approach did not cater for the heterogeneous IoT system as it made assumptions that all nodes had the same level of energy storage. Although most edge nodes have a longer lifespan than sensor nodes, they, like the sensor nodes, are also plagued with connectivity and energy consumption problems, similarly owing to their rechargeable battery constraints.

It is to this effect that this paper aims for further better resource optimisation for the heterogeneous IoT by integrating concepts of EC and clustering as well as a hybrid algorithm consisting of the static round robin (RR) and the dynamic resource-based (RB) algorithms. Amalgamating these concepts will aid in re-configuring IoT’s central topology into a decentralised topology, thereby mitigating the problems associated with energy consumption and quick death of nodes. This approach, taking into consideration the varying energy storage and computational capacity, will also lessen the workload of all the nodes within a cluster, thus ensuring energy dissipation is managed. The CH will be saddled with the responsibilities of providing the shortest path for resource allocation as well as offloading excesses to the cloud, thus guaranteeing the CH’s energy consumption is considerably lowered, consequently evading overall system performance degradation. The major contributions of this article are

1. An improved there-tiered framework for efficient resource optimisation in IoT. The framework, as opposed to existing frameworks, performs resource optimisation by placing equal importance on resource scheduling and resource allocation.
2. A mathematical model incorporating the proposed framework. This model following the proposed framework takes into consideration the heterogeneous nature of IoT devices and harnesses their distributed nature to reduce nodal energy dissipation, subsequently prolonging the system’s lifespan by mitigating the quick death of nodes.
3. An analysis of the simulation, depicting the effectiveness of the proposed model. The model’s ability to perform adequate load balancing generates results that illustrate its efficacy. Comparisons made with the current state of the art illustrates the superiority of the proposed model.

The rest of the paper is organised as follows: Section 2 discusses related works, providing further insight into the motivation behind this paper, and Section 3 delivers the proposed model and model algorithm. Section 4, on the other hand, presents and applies the proposed model to resource allocation in edge nodes of the IoT system; in this section, analysis are made validating the effectiveness of the proposed model via simulation experiments and comparisons between the proposed model and other intelligent optimisation algorithms. Finally, Section 5 concludes the paper.

## 2. Related Work and Motivation

This section further intricates related works and considers the lapses that need addressing. The section also contains the resource optimisation problem and motivation for the proposed model.

### 2.1. Related Work

IoT’s increasing popularity equates to the incumbent surplus of data generation that requires competent management schemes. Equally, with an increasing number of IoT devices connecting to the cloud, users’ expectations and demands for high-quality services have heightened. To efficiently meet these expectations that hinge on solutions of the prevailing RAPs, recent work has shifted focus to issues of the EC paradigm that may affect the effectiveness of service provision [16]. IoT’s heterogeneity and difference in performance rates necessitate mechanisms for efficiency. Software-defined networks (SDN) mostly offer the EC paradigm adequate solution for RAP by enabling it to run IoT applications with minimum end-to-end delays [3]. EC’s capability to extend cloud computing services to the edge of the network, which makes it a plausible solution for IoT applications, and, increasingly, EC is being used to optimize resource scheduling in IoT. Similarly, in IoT, clustering algorithms in conjunction with EC have proven to be effective procedures for lowering the energy dissipation of nodes, alleviating the sensing coverage problem, balancing the energy consumption among nodes, and prolonging the network lifetime in contrast with flat routing algorithms [12,13]. Clustering, an unsupervised machine learning technique allowing for the grouping of nodes that run in parallel to achieve set goals, is of various types: density-based, where grouping is considered based on concentration of nodes; distribution-based, in which nodes are considered parts of clusters pending on the probability of belonging to that cluster; centroid-based, wherein nodes are assigned to clusters based on their distance from a centroid; and hierarchical-based, which builds a tree of clusters. Clustering consists of two major stages: the formation of clusters, and the cluster operation. During the formation of clusters, all nodes are organised into groups based on set parameters and a cluster head (CH) is designated; this designation can be fixed, leading to a permanent CH per cluster or variable where multiple nodes have an equal probability of becoming the appointed CH within a cluster pending on set parameters. The incorporation of EC and clustering algorithms as solutions for RAPs implies a paradigm shift, and with this comes the terrain of unstable CH selection and energy consumption amongst clusters. Ref. [13] provides a summary of several tactics for solving the unstable selection of CH problem; these strategies include clustering methods; the genetic algorithm; combining the genetic algorithm and the probability forwarding criterion; and combining the characteristics of genetic optimisation and routing in LEACH (low-energy adaptive clustering hierarchy). A number of other works put forward various approaches for CH selection; these approaches include the cluster-head selection method based on a node’s residual energy, the Dijkstra routing strategy, the hybrid energy-efficient distributed unequal clustering algorithm, and rotated unequal clustering algorithm for wireless sensor networks [13,17]. Optimizing the size and composition of the cluster is dependent on several characteristics: nodal residual energy, the position of cluster heads, the link status, and many more. Although the advantage in finding the best solution is obvious, disadvantages associated with CH selection, specifically in CH rotation, include a great computational overhead and multiple complexities that further mar the current state of the art.

Clustering, an efficient topology control method, which balances the traffic load of the nodes consequently, improving the scalability and lifetime of system, has a lingering problem that may lead to quick death of the CHs, thereby degrading the overall performance of the system [18]. When only one CH exists in the cluster, the nodes around the CH consume their energy quickly, and if not controlled quick death is inevitable. The quick death of CHs arises due to higher energy consumption necessitated by the CHs’ extra workload of receiving, aggregating, and transmitting data to the nodes, other CHs, and resources as the need may arise. The improper formation of clusters can also cause some CHs to be overloaded with a high number of nodes [2,18]. In [15], comparisons were made between seven (7) existing algorithms, namely, LEACH (low-energy adaptive clustering hierarchy); HEED (hybrid energy-efficient distributed); DWEHC (distributed weight-based energy-efficient hierarchical clustering protocol); PEGASIS (power-efficient gathering in sensor information systems); CCS (centralised clustering algorithm); balanced energy-efficient (BEE); sensor web (S-WEB); and direct algorithm (non-cluster routing solution). Of the seven (7) algorithms, all but the S-Web and direct algorithm were infused with CH rotation as a measure to reduce any form of nodal failure—especially the quick death of CHs, and all but the direct algorithm were clustering algorithms. Despite measures to reduce the impact of node failure via CH rotation mechanisms in the LEACH and HEED algorithms, the generated results of comparing the LEACH, HEED, and direct algorithms indicated miniscule improvements in the number of sensor nodes still alive in the network after several rounds, especially for larger rounds. The authors of [19], in their work, proposed the S-web algorithm, which was compared to the direct algorithm; in their comparisons of the two algorithms, small improvements were also seen as S-web performs slightly better than the direct algorithm. This suffices to say and further proves that clustering algorithms when not properly implemented can be redundant in functionality and even when included in design processes; focusing on CH rotation alone is not enough as there are several other factors that directly or indirectly affect the CH energy dissipation and subsequently the network lifespan. Controlling other factors such as communication and computational overhead; the size of the cluster; the path length between nodes and resources; and CH activities, that is, the responsibilities the CH is saddled with, may in fact yield better results in terms of bottle neck and lifespan of the network. The authors of [18] also investigate several clustering algorithms and make comparisons of the effectiveness of such algorithms against their proposed differential evolution (DE)-based clustering algorithm called DECA, which was designed with the main objective of prolonging network life by taking care of the energy consumption of nodes and cluster heads (gateways). Algorithms that [18] compared to their existing algorithm include GLBCA (greedy load balancing clustering algorithm), LBC (load balanced clustering), EELBCA (energy-efficient load-balanced clustering algorithm), traditional DE (differential evolution), and GA (genetic algorithm). Comparisons were again made on the grounds of network lifespan, comparing the balancing lifetime of gateways, the number of dead nodes per round, the energy consumption per round, and the convergence rate. Ref. [18]’s experimental results show that their proposed algorithm converges faster than the traditional DE and GA and performs better than the exiting algorithms, that is, the traditional DE, GA, LBC, and GLBCA in terms of network life, energy consumption, and number of dead sensor nodes; however, it performs worse than the other existing algorithm, namely, EELBCA, in terms of energy consumption and the number of dead sensor nodes. In all the comparison cases made by [18], no references or data were generated to implore the effects their modelled algorithm had on the overall system’s performance in terms of the system’s efficiency measured by the system’s turnaround time.

Controlling the problem associated with the quick death of nodes, especially CHs, will make room for the advantages associated with cluster-based systems to further impact the optimisation capacity of any given system. Cluster-based systems have multiple advantages, which make the resource optimisation of IoT systems necessary; such advantages include the discarding of redundant and uncorrelated data, thereby reducing energy consumption by nodes within the cluster; the easy management of routing, resulting in improved scalability; and the conservation of communication bandwidth. Clustering algorithms are also beneficial for the improvement of energy efficacy and a reduction in transmission delay [13]. The clustering of nodes has proven to be an effective solution for prolonging the network lifetime, which is a primary key to measuring system’s performance. The approaches for CH selection as mentioned in [13,17] can be grouped into two major CH selection schemes: the homogeneous schemes in which all the nodes are initially equipped with the same amount of energy, and the heterogeneous schemes, where all the nodes are equipped with the different amount of energy due to their varying functionality [20]. Given the heterogeneity of IoT, the heterogeneous scheme will govern the CH selection type in the proposed model. The clustering type considered is the centroid-based where parameters of contention are CHs distance from the servers and subsequently an edge node’s distance from the CHs. The distance from the centroid is considered due to its sensitivity to given parameters, speed, and efficiency, unlike other approaches, where several more parameters come into play. Thus, the direction of development aims to improve the stability/robustness and convergence rate of EA [1], specifically PSO. For a better grasp of the underlying motivation behind the proposed model, a theoretical analysis of PSO, round robin (RR), and resource-based (RB) algorithms will steer into the right direction for solving resource optimisation problems in IoT, placing equal importance on resource scheduling and allocation.

#### Overview of the PSO, RR, and RB Algorithms

Particle swarm optimisation (PSO), a stochastic optimisation technique based on the movement and intelligence of swarms, is an increasingly popular algorithm for solving resource-scheduling problems owing to its simplicity in operation and speed in convergence [1]. PSO, also considered an EA due to its use of mechanisms motivated by nature, starts from a random solution and determines the optimal solution by iteration; previously good positions attract the particles, and evolution takes place if a particle flies to a better contour [2,10]. PSO has been found to be superior to GA considering that PSO carries global and local search simultaneously, as opposed to GA concentration on just the global search. Although PSO has numerous advantages and has presented outstanding performance in solving practice optimisation problems, IoT inclusive, it still has inherent defects that make it prone to falling into local optimum, premature convergence, and excess overhead cost [1]. When discussing the evolution of the particle swarm, the search space instead of the problem space is of concern; each particle’s position can be regarded purely as a D-dimensional vector, and for a single point in the D-dimensional search space (D = 2T), similarly, each particle’s velocity is a D-dimensional vector [10]. The particle-swarm algorithm can search a large candidate solution space without gradient information. The PSO algorithm is simple in structure and fast in convergence, which is suitable for scheduling; however, the PSO is prone to the local optimum, with fast convergence necessitating that the global search be performed first. When an optimal solution is determined within a specific range, the local search ability is used to search for the optimal solution position [21]. Given the PSO’s unassuming and easy implementation characteristics, it is used to solve the problem of resource optimisation in IoT.

An effective way of mitigating and or completely avoiding data collision is to introduce some communication protocols to schedule the data transmission [22]. The round robin (RR) static algorithm, the oldest and simplest scheduling protocol or algorithm, which is usually used for multitasking, allows for equal time slot allocation of tasks/requests to resources/servers. Each task runs turn by turn pending on the cyclic queue bound by a time slice also referred to as quantum time [22,23]. Round robin is a real time pre-emptive algorithm that responds to real-time events; at each transmission instance, the round-robin regulates a node’s access pending a predetermined circular order [23].

Resource-based (RB) algorithms, on the other hand, use a heuristic methodology by means of a greedy approach, prioritizing the allocation of resources from the largest to the lowest [8]. That is, it iteratively selects the most demanding request/task and allocates it an appropriate and available resource/server for processing, thereby maximizing throughput and reducing power [8,9]. The RB dynamic algorithm is practical for heterogeneous requests/tasks, where records of performance of the resources over a period are accessed for finding the best match; this implicates the overall performance improvements.

The dynamic RB algorithm’s ability to adapt gives the RR-RB hybrid algorithm the opening of becoming a controlled adaptive algorithm for load balancing and consequently the resource optimisation of IoT systems. The deficiency of falling into the local optimum experienced by the PSO algorithm is tackled in the proposed model by the incorporation of the RR-RB hybrid algorithm. This meld ensures the best path is always presented as the best of both worlds is deployed, a cluster that is both efficient and void of quick CH death due to excess energy dissipation, thus allocating resources, balancing load efficiently, and prolonging network lifespan.

### 2.2. Resource Optimisation Problem and Motivation

This subsection delves into the problem formulation; it additionally expounds the motivation for developing the simulation.

#### 2.2.1. Resource Optimisation Problem Statement

IoT’s amalgamation of diversified devices implies a varying assortment of activities ranging from sleep monitoring to the tracking of activities. Devices people wear and use are becoming increasingly sophisticated, being able to connect to media accounts and track data, thereby helping enrich lives; however, with such activities comes ample data transfer and storage problems. With a vision of an all-communicating world, the number of connected devices and the user data-rate is to be increased by ten to a hundred times; the extended battery life by up to ten times for massive machine communication devices; and end-to-end latency by five times [4]. The IoT’s current topology gives rise to increased complexities, with just an increase, in the form of an added node, of complexities such as scalability, latency, and compatibility increase exponentially, bringing about underrated services. The resource optimisation problem can be divided into two main categories: the resource allocation problem and the resource scheduling problem. A plethora of works have aimed at the optimisation of IoT; however, such works either focus on the allocation of resources to the detriment of resource scheduling and vice versa or are domain-specific. Domain-specific works, for instance, include the works that focus on task scheduling and/or task management in the health sector [24,25,26], or on task offloading and scheduling in transport applications [27], or even on industrial automations [3]. It therefore suffices to say that a generalised IoT framework applicable in all IoT scenarios is yet to be fully accepted and implemented. Resource optimisation is a huge step in alleviating complexities associated with prevalent IoT systems, and the right approach can make IoT systems more efficient and complacent to users’ expectations. To solve the abovesaid challenges, this paper proposes a novel resource optimisation algorithm that will be applicable in various IoT environments.

#### 2.2.2. Motivation and Lapses

The value-added services and real time applications of IoT come with ample data generation and consumption; therefore, the major problems that require great attention are multidimensional optimisation problems, where concerns involve determining in real-time how to select the optimal service configuration and provide efficient edge service scheduling scheme [1]. Additionally, the resource under-utilisation problem and high operational cost of IoT-based cloud system noticed in the dynamic resource allocation (DRA) technique [6] are other areas of concern. Decentralising IoT’s topology comes at a detriment to security, and this lapse is further amplified by the edge nodes’ frail capacity in terms of storage and power. To be able to bypass these lapses, an efficient resource optimisation scheme, placing equal importance on resource allocation and scheduling, is needed. Additionally, the effectiveness of the algorithms to meet users’ exacting standards (QoE and QoS) and edge nodes—especially sensor nodes life span are other vital areas of concern that require a breakthrough.

The use of the EC paradigm increases security and quality assurance as it encourages the processing of data closer to source of generation, thus reducing the number of data transferred between a device and centralised node. The work of [6] shows the improvements of a hybrid algorithm combining a static and dynamic algorithm have on resource allocation; our previous work shows how the hybrid load balancing approach, a merger of the EC paradigm and RR-RB hybrid algorithm, produces an effective load-balancing strategy for resource optimisation in IoT. This paper takes the optimisation technique a step further by incorporating aspects of clustering, as appropriate use of clustering techniques inadvertently reduces the latency by half through enabling IoT devices to fulfil complex analysis tasks with lower latency, higher performance, and less energy consumption [9,16]. To further answer the question: what computational and algorithmic theories are suitable, in practice, for management and resource optimisation of internet of things?, this paper proposes an algorithm that comprises an amalgamation of aspects of the EC paradigm, the particle swarm optimisation clustering technique, and an RR-RB hybrid algorithm to produce an algorithm tagged PSOR2B for the purpose of solving resource allocation and scheduling issues in IoT, thereby delivering an efficient resource optimisation technique in the IoT environment that is comparatively secure.

## 3. Proposed Solution

This section presents the proposed model’s description in addition to the model’s algorithm. The focus is on the algorithms functionality in achieving resource optimisation for IoT.

### 3.1. Model Description

**Figure 1:**
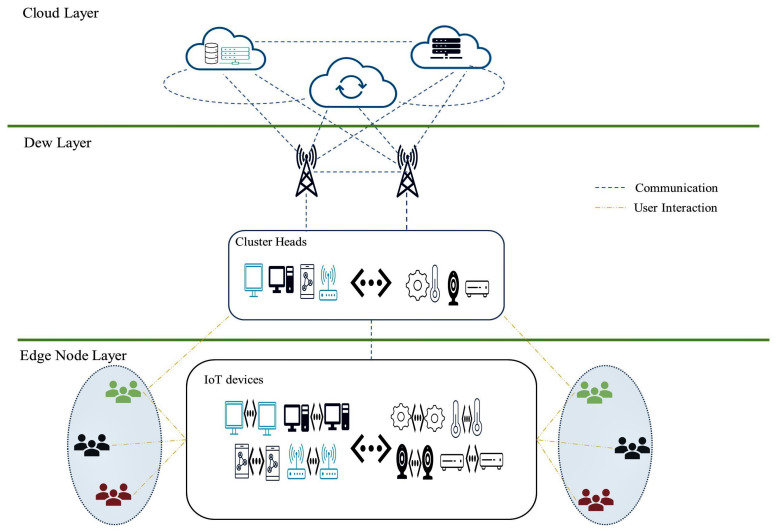
Proposed model.

The architecture of the proposed model in a bottom up fashion, as portrayed in Figure 1, is divided into there (3) layers: edge node layer, dew layer, and Cloud layer. The edge node (first) layer comprises of different IoT device with varying computation, processing, and storage capacity. The devices in this layer are clustered based on their proximity to the CH found at the dew (second) layer. The clustering is governed by the PSO algorithm, and edge nodes can acquire services from the dew and cloud layers. The dew (second) layer made of the CHs and edge servers is saddled with the task of resource allocation and scheduling. The CH selected based on its Euclidean distance to the edge server and relative storage capacity as well as its computational power utilizes the hybrid algorithm tagged RR-RB (round robin—resource-based) to efficiently schedule and allocate resources to the appropriate Edge nodes once a request is initiated. The CHs tackle latency and redundancy by creating and deleting paths of frequency and recency after consideration of server rates. The formulated paths ease the allocation and scheduling of resources once a request/task is initiated by an edge node or in some cases the CH itself. Excess data are stored in the edge server as well as cloud servers. By creating a record of updated paths of frequency and recency for effective scheduling and allocation of resources, CH’s workload is reduced. This therefore manages its processing workload, adequately controlling computational overhead. Furthermore, the EC paradigm enables the CH to efficiently reduce data transfers required between itself and requesting nodes, and CH’s ability to store excess data in the edge or cloud servers consequently mitigates rising energy consumption. The cloud (third) layer consisting of cloud servers acts as a resource as well as a repository. The CH pending on former performance is aware of each servers’ processing capacity in relation to requests made by the edge nodes and can adequately allocate resources to tasks/requests initiated by nodes.

### 3.2. Model Algorithm

The proposed model intends to optimize resource allocation by incorporating aspects of the RR-RB hybrid algorithm, EC paradigm, and clustering (particle swarm optimisation) technique. The trio tackle the aforementioned complexities that come with IoT by scheduling and allocating resources to provide a solid foundation for an optimised and secure IoT. The trio algorithm accomplishes this by working together to efficiently bundle the edge nodes into clusters, designating a CH, and taking into consideration the resource/server rates and task/request size, thus sustaining set parameters. The scheduling and allocation processes require taking decisions, and the decision makers are comfortable in using Pareto-optimal solutions because if the final solution is not Pareto-optimal, it can be improved in at least one objective without deteriorating the solution quality in other objectives [7,12,13].

The allocation of resources is governed by the PSO as well as aspects of the RR-RB algorithms. The PSO determines the motion direction of each node by a kinetic equation that includes two fundamental properties: velocity $v_{i}$ and position $x_{i}$. The clustering of the edge nodes is affected by three types of information: individual inertial velocity, global best position, and individual current history best position. Each node is assessed by its fitness function f(x) containing three vectors and a global vector [1]. The fitness function plays a crucial role in the algorithm’s performance. Equations (1) and (2) represent how the clustering is affected by the three types of information.

(1) $$v_{i}\left(t+1\right)=wv_{i}\left(t\right)+c_{p}r_{1}(b_{i}\left(t\right)-x_{i}\left(t\right))+c_{g}r_{2}\left(g\left(t\right)-x_{i}\left(t\right)\right)$$

(2) $$x_{i}\left(t+1\right)=x_{i}\left(t\right)+v_{i}\left(t+1\right)$$

(3) $$b_{i}\left(t\right)=x_{i}\left(t_{p}\right):f\left(x_{i}\left(t_{p}\right)\right)=\min_{0\leq k\leq t}f(x_{i}\left(t_{k}\right))$$

(4) $$g\left(t\right)=b_{ib}\left(t\right):f\left(b_{ib}\left(t\right)\right)=\min_{0\leq i\leq N}f\left(b_{i}\left(t\right)\right)$$

where $x_{i}\left(t\right)$ is the *i*th particle’s position in the *t*th iteration; $v_{i}\left(t\right)$ equals the *i*th particle’s velocity in the *t*th iteration; $b_{i}\left(t\right)$ equates to the *i*th particle’s history best position, which has visited until the *t*th iteration; and *g*(*t*) is the particle swarm’s global best position of the *t*th iteration. Additionally, the key control performance parameters of the algorithm are *w*—inertia weight constant, used to control the impact of $v_{i}\left(t\right)$ in the process of the evolution of particles; the $c_{p}$—cognitive coefficients, which pull particles toward $b_{i}\left(t\right)$; and $c_{g}$—social coefficients, which pull particles toward *g*(*t*). The RR-RB, on the other hand, further buttresses the allocation by routing requests/tasks to available resources/servers, making certain that the tendency of falling into local optimum is avoided.

The scheduling of resources governed by aspects of both the RR and RB algorithm ensures that all requests obtain an economical allocation of resources, that is, ensuring that requests/tasks are allocated a time slice/quantum time according to their size without prioritising requests, by this means providing the best performance in terms of average time. The hybrid avoids starvation or convoy effect, that is, slowing down because of several time-consuming processes. Finally, the hybrid allows for the allocation of requests to resources based on request size and resource capacity. This allocation criterion further improves resource optimisation for IoT. The quantum time is taken into consideration when scheduling resources. Let: *Q* signify the quantum time, that is, the time the scheduler allows for a task to run; *TTS* the total task size coined by the edge nodes for execution; and *TR* the transfer rate. If the quantum time of the *i*th task in the *j*th resource is *Q*(*i*, *j*), the task size is *TS*(*i*, *j*), and the transfer rate is *TR*(*i*, *j*), then the turnaround time (*TAT*) can be expressed as:

(5) $$TAT=\frac{TS(i,\;j)}{TR(i,\;j)}\times Q\left(i,\;j\right)$$

where *Q*(*i*, *j*) is defined as:

(6) $$Q=\;\frac{TTS(i,\;j)}{TR(i,\;j)}\;\;$$

and *TTS* is the total task size, that is, the summation of all tasks’ (Σ*T*) awaiting resources.

**Figure 2:**
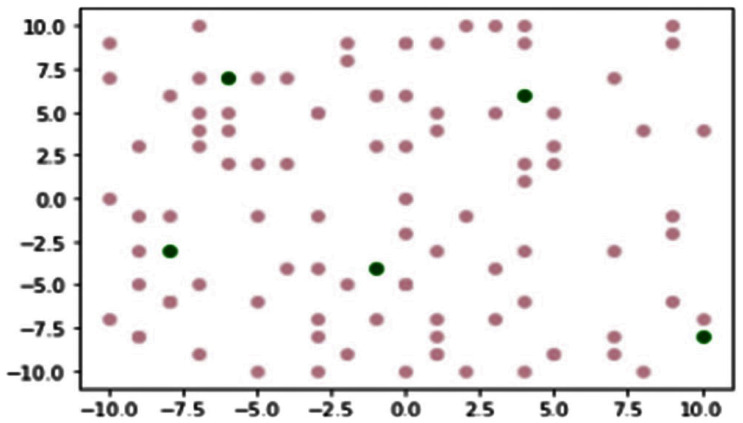
Cluster creation.

Typical PSO clustering algorithms form clusters by allowing the nodes to join CHs based on locality, assuming that nodes are equally distributed. If the formation of clusters allows nodes to join the nearest CH, then the CHs of densely deployed areas will be overloaded with higher number of member nodes [13]. The proposed model tackles this problem by creating clusters and consequently CH based on the criterion of locality and the upper limit of nodes allowed within a cluster. CH are designated based on the nodes’ proximity to the resource/server; this, therefore, implicates that the number of CHs greater or equal to (≥) the number of resources/servers; once the CH is established, the nodes join the CH based on their proximity to the designated CH, with the closest being first considered till the upper boundary for number of nodes within each cluster is met, thereby creating almost equal sised distributed clusters. The upper limit is set after taking into consideration the number of edge nodes and CH. The CH’s proximity to the server inadvertently reduces energy dispensation, by this means prolonging lifespan of the CH node. Figure 2 depicts how the clusters and CHs are created: the pink dots represent edge nodes, while the green dots represent resources/servers. In this Figure, tentatively, based on proposed parameters, the number of clusters/CH is greater or equal to (≥) five (5) and the upper limit for number of nodes within the cluster is twenty (20).

A plethora of recent works on the PSO algorithm focus on using PSO as a means of finding the appropriate number of clusters according to data characteristics; the average trajectory and velocity of nodes; the time cost of nodes; the energy consumption of CHs; the lifetime of nodes; and the total residual energy of nodes [1,13,17,18]. Harnessing the key advantages highlighted in these works, the proposed model clusters and initialises the edge nodes based on Cartesian proximity to servers. This action gives rise to an end product that resembles a more realistic system, posited to positively affect propagation, consequently paving way for the hybrid algorithm to efficiently function. The output at the end is an efficient and novel load balancing strategy that allows resource optimisation for all IoT systems. Based on the criterion of proximity to the server/resource, the initialisation of clustering regulated by the PSO classifies the nodes into two major categories: cluster heads and edge nodes. This clustering hinges on the physical topology of nodes and consequently IoT. It is presumed that the number of CHs and severs are known; however, in reality, a server represents a myriad of servers. The load balancing, and consequently the resource optimisation, is regulated by the trio algorithm, and, at the dew layer, the cluster heads make use of the hybrid algorithm alongside the PSO algorithm; the combination of these algorithms allow for the efficient flow of the proposed model and subsequent optimisation of resources in IoT.

Unlike flat routing algorithms, the proposed model as depicted in Figure 1 is structured in a hierarchical, thereby improving scalability. With over fifteen (15) billion devices out of the twenty-eight (28)-plus billion smart devices connected across the world employing machine to machine (M2M) communication [4], there is a rising need for systems to be implemented in ways that allow for such communication modes with ease; consequently, in the proposed model, M2M as well as machine to people (M2P) are the conceivable modes of communication. The first layer tagged, the edge node layer, contains all of edge nodes; these edge nodes, all IoT devices, are grouped in clusters answerable to the CHs. At this layer, the mode of communication is machine to machine (M2M) and machine to people (M2P). The second layer, referred to as the dew layer, contains CHs and edge servers and at this layer; the major mode of communication is M2M and extremely limited M2P, that is, in the case where the CH has a task of its own triggered by end users to complete, finally at the third layer, denoted the cloud layer, the only mode of communication is M2M.

**Figure 3:**
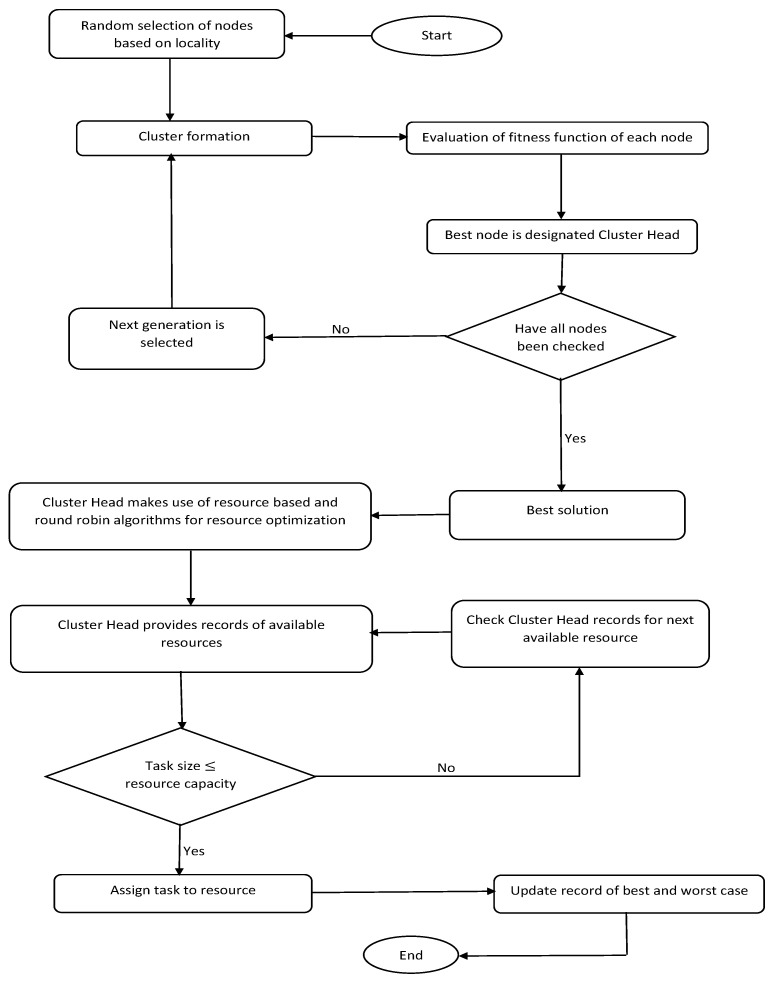
Model flowchart.

Algorithm 1 is an algorithm showing how PSO works in the proposed model, and the flow chart represented in Figure 3 is a pictorial representation of the steps the proposed system takes to achieve resource optimisation in IoT. In Algorithm 1, the edge nodes generating tasks are referred to as particles, and in the proposed model’s case scenarios, the initial position of edge nodes (particles) is randomly generated. Once the initialisation phase elapses, the mapping of the updated position; the obtaining of the fitness value; and the updating of the velocity, position, and fitness function of all of the edge nodes (particles) are conducted. After the particle’s position has been updated using (2)–(4), the new position is compared to its incumbent updated position. The updated position is replaced with the new position if the updated position is greater than the new position; otherwise, maintain the updated position if vice versa. The global best is determined by comparing the local best, that is, the optimal solution of a given particle to the global best—the optimal solution of all particles. If a particle’s position satisfies (5) and (6) by obtaining the fitness value and ensuring that the local best equals the global best, it is successfully allocated to a cluster; otherwise, it is left in the pool for further allocation to an appropriate cluster. As (5) and (6) are both linear, it is convenient to convert infeasible positions to feasible positions by adopting a repair strategy [10].
**Algorithm 1** PSO Algorithm for proposed model. 1: Initialize position $x_{i}\left(t\right)$ and velocity $v_{i}\left(t\right)$: 2: Check particle’s position and velocity 3: Evaluate current fitness 4: Mapping of the updated position to the corresponding particles adjusting minimum and maximum position if necessary 5: Obtain the fitness value of each particle 6: if $b_{i}\left(t\right)=g\left(t\right)$ 7: Update the velocity $v_{i}\left(t\right)$ of each particle 8: Update the position $x_{i}(t$) of the particle 9: Update the fitness f(x) of each particle 10: Repeat Step 5 to Step 9 until it reaches maximum number of iterations is reached. 8: Particle with position closest to resource is designated cluster head 9: Cluster head broadcasts its position to adjacent nodes and completes cluster creation


Assume the set of edge nodes are denoted by N = $\left\{n_{1},\;n_{2},\ldots ,n_{num}\right\}$ and $CH_{n}$⊆ N, the set of CHs denoted by CH = $\left\{ch_{1},\;ch_{2},\ldots ,ch_{num}\right\}$ has $E_{ClusterHead}\left(ch_{i}\right)$, and the total energy of $ch_{i}$ and residual energy $E_{residual}\left(ch_{i}\right)$ is the remaining energy of $ch_{i}$ in a single round. The CHs’ total energy consumption in a round is dependent on the number of nodes within its cluster. If $ch_{i}$ has $num_{i}$ number of nodes, then its total energy consumption is: $E_{ClusterHead}\left(ch_{i}\right)$ = $num_{i}\times E_{R}+num_{i}\times \;E_{DA}+E_{T}\left(ch_{i},S\right)$, where $E_{R}$ is the energy consumption concomitant of receiving requests/data, $E_{DA}$ is the energy consumption due to data aggregation, and $E_{T}$ is the energy consumption due to data transmission. Given that each CH is battery powered and has a tendency to experience downtime, the lifetime of a CH represented by $L_{i}$ can be calculated as follows: $L_{i}$ = $\frac{E_{residual}\left(ch_{i}\right)}{E_{ClusterHead}\left(ch_{i}\right)}$.

## 4. Experiment and Analysis

This section delivers the simulation setup and analysis of results acquired. The simulation setup describes the approach taken to evaluate the efficiency of the proposed model, and the analysis of results acquired from the simulation setup will give insight as to the efficacy of proposed PSOR2B model.

### 4.1. Experimental Simulation Setup

The parameters and dataset remain the same in all test scenarios. The simulations were on one to one hundred (1–100) nodes and five (5) servers, thus reflecting corporeal situations where the edge nodes are multiple in comparison to the limited number of resources and buttressing the requirement for the management of limited resources. The nature of the model, which simulates a real-life situation, implies that all nodes have varying battery energies and are randomly deployed; the message and packet sizes vary with each request; the mode of communication is bidirectional—wireless, or not, M2M, and M2P; and the mode of communication to cloud and edge servers is wireless—M2M. In the simulation run, the simulation parameters as shown in Table 1, similar to aspects of [13,17,18], were used.

Five (5) test case scenarios of the proposed model were simulated using the python programming language and the anaconda framework. These test case scenarios were initiated to evaluate the performance of the proposed mode are grouped into preliminary and secondary test scenarios. The preliminary scenario, which equates the first three test case scenarios, is concerned with establishing the relevance of the PSO algorithm. The secondary test scenario, which includes the last two test scenarios, is concerned with portraying PSOR2B’s efficacy over existing approaches. The scenarios enabled one to measure the effectiveness of the model as a means for the further optimisation of resources in IoT systems. The performance of the proposed model is assessed based on the returned turnaround time (*TAT*). The proposed model leveraging on the sever rates aims to balance resource allocation, consequently improving the server rate by allocating appropriate resources/servers to request/edge nodes. The *TAT* performance indicator, which is the amount of time it takes to complete a given process, is a worthy measure of the effectiveness of the proposed model as in effect it is the measure of the server/resource rate. As previously mentioned, the turnaround time (*TAT*) is expressed as Equation (5).

### 4.2. Experimental Results and Analysis

Related works indicate that the appropriate use of clustering algorithms produces better solutions as opposed to non-clustering algorithms; hence, at the preliminary phase, three test scenarios were conducted. The first test scenario compares the efficiency of two clustering algorithms—PSO contrasted with Ant colony. The existing ant colony implementation found in [28] was chosen as its areas of focus match the areas of focus of the proposed system, Moreso; comparing the two will bring enlightenment and inform the decision regarding the choice of clustering algorithm applied to the proposed model, thus championing the incorporation of the best clustering algorithm into the proposed model as well as the best course of action for resource optimisation of IoT.

**Figure 4:**
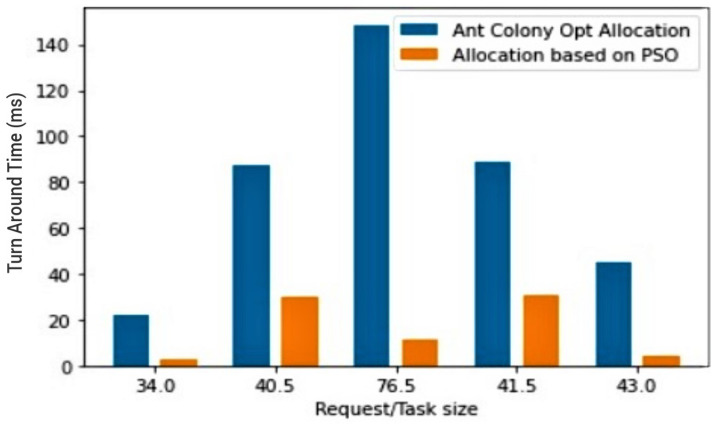
Ant Colony vs. PSO clustering technique.

Figure 4 depicts without a doubt PSO’s great advantage over the ant colony algorithm when it comes to the efficiency of resource allocation in IoT; the larger the task, the greater the efficiency. This reiterates its flexibility and scalability and implies that aside form its efficiency, PSO complies with the proposed systems’ objective, enhancing its resource optimisation and improving its scalability.

**Figure 5:**
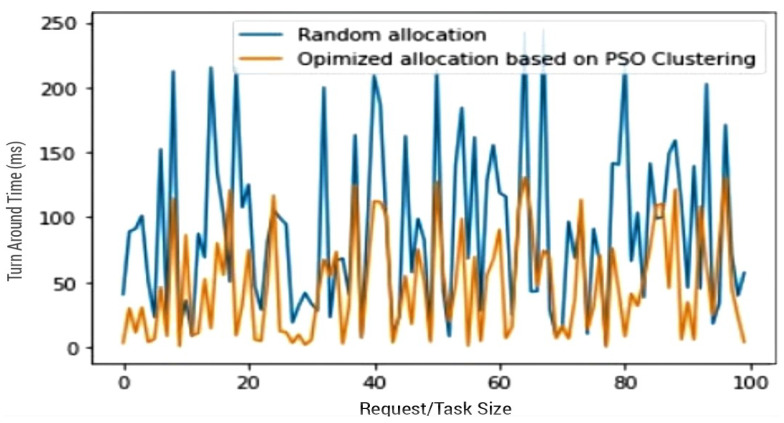
Random allocation vs. PSO clustering technique.

After the establishment of PSO’s alignment with the proposed model’s objective, and its superiority over the ant colony clustering algorithm, the second preliminary test conducted set out to measure the efficacy of the PSO clustering algorithm as an algorithm for resource optimisation in IoT. Figure 5 demonstrates how clustering (PSO) stands to cause improvements on resource optimisation techniques. The random allocation of resources, as employed in a centralised IoT, produces a much higher *TAT* than the PSO clustering algorithm. Clustering (PSO) was applied, and evident improvement over the random allocation can be seen. This result not only illustrates the improvements clustering has on resource optimisation in IoT, it also reaffirms the decision of incorporating PSO in the proposed model as it justifies the necessity for the use of clustering as an additional means of resource optimisation in IoT technologies.

The first two preliminary test cases establish the dominance of PSO over Ant Colony and PSO’s effectiveness over random resource allocation; the aforementioned therefore aided the proposed model’s design. Assumptions made in the model’s design regarding the clustering of nodes and consequently the creation of CHs is that the server’s performance is a function of proximity to request on a Cartesian plane; thus, the CH and subsequent cluster formation was dependent on their proximity to servers, with the CH being the closest with favourable storage and processing capacity.

**Figure 6:**
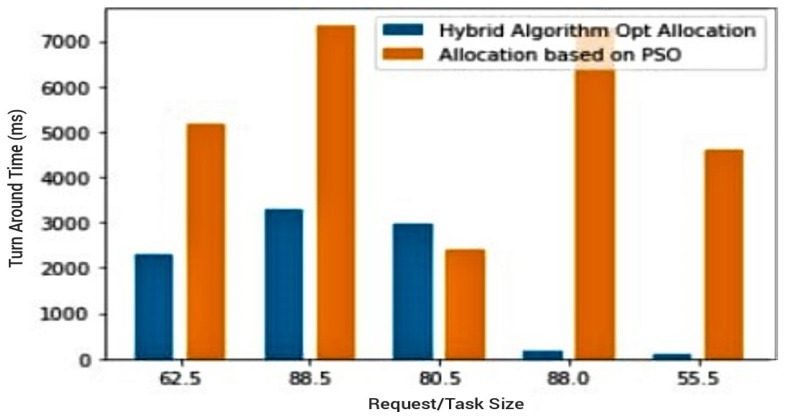
Hybrid resource allocation algorithm vs. PSO clustering technique.

The third preliminary test scenario looks at the effects of the previous proposed hybrid algorithm contrasted against the PSO has on resource optimisation. Having established PSO’s ability to optimize resource allocation effectively as depicted in Figure 5, the RR-RB hybrid algorithm was subsequently compared to the PSO as a measure of its efficiency. The output as represented in Figure 6 shows that the hybrid has dominance over the PSO approach. The results displayed on the graph show how the hybrid algorithm’s *TAT* is over three times more efficient than that of its counterpart the PSO. This third instance proves the advantage of using the hybrid in the proposed model, and although PSO has multiple advantages and has better efficiency than the ant colony and random allocation algorithms, its efficiency is limited and can be further heightened.

**Figure 7:**
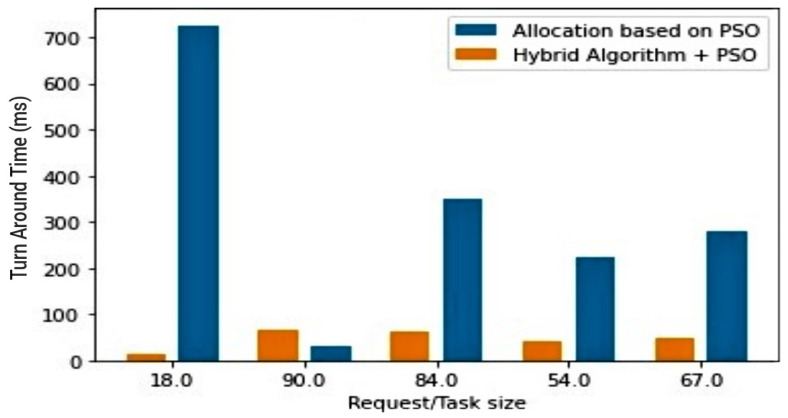
PSO vs. PSOR2B (Barchart).

**Figure 8:**
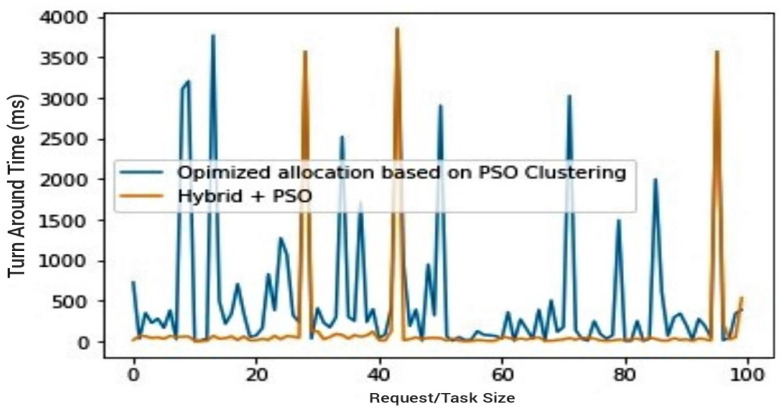
PSO vs. PSOR2B.

**Figure 9:**
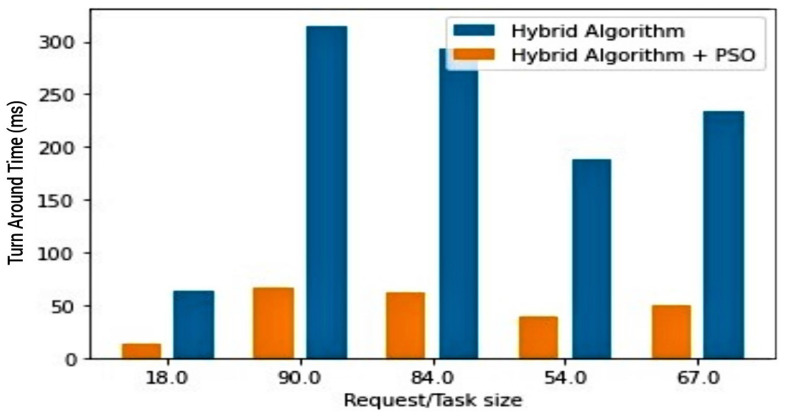
Hybrid vs. PSOR2B (barchart).

**Figure 10:**
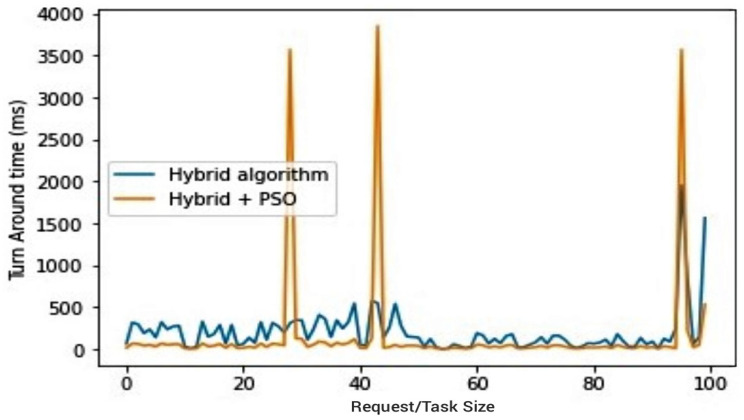
Hybrid vs PSOR2B.

**Figure 11:**
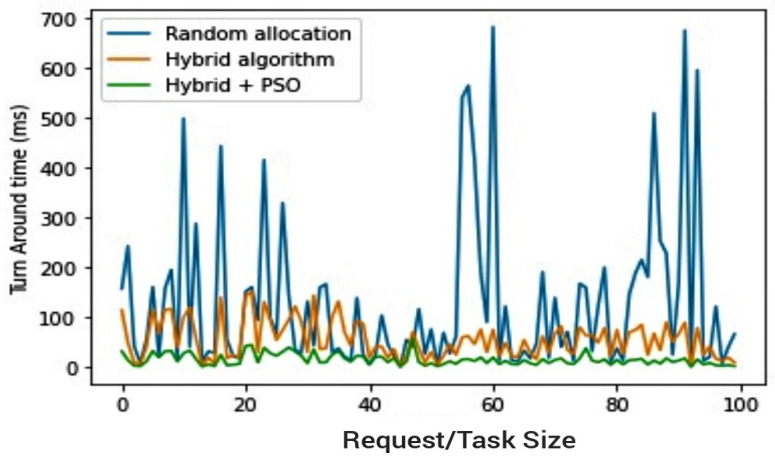
Random resource allocation vs. hybrids resource allocation vs. PSOR2B.

**Figure 12:**
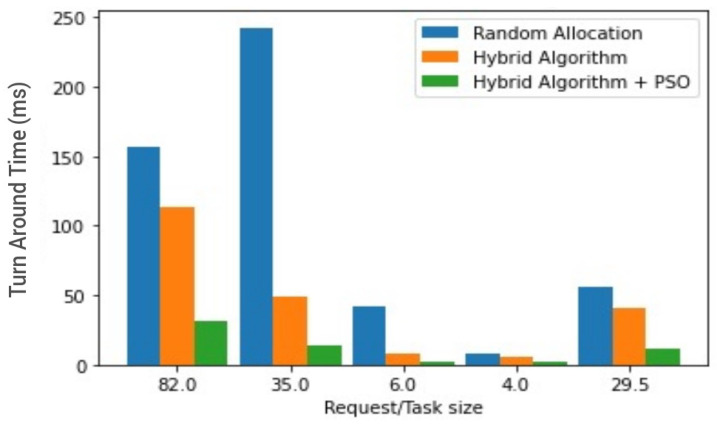
Random Resource allocation vs. hybrids resource allocation vs. PSOR2B (Barchart).

**Figure 13:**
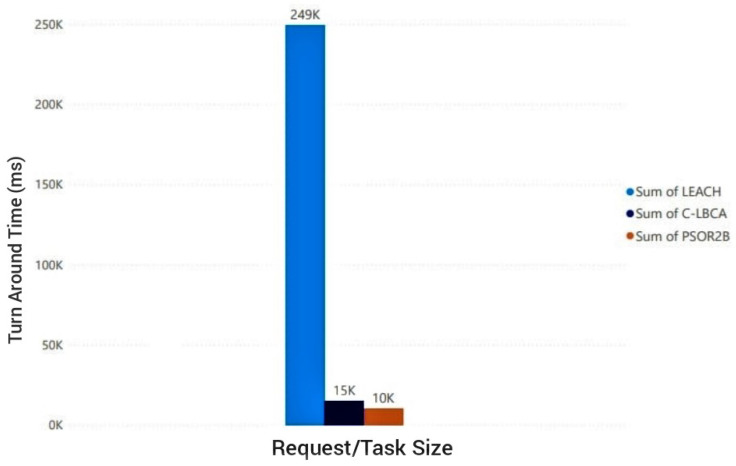
Cumulative turnaround time of LEACH vs C-LBCA vs PSOR2B.

**Figure 14:**
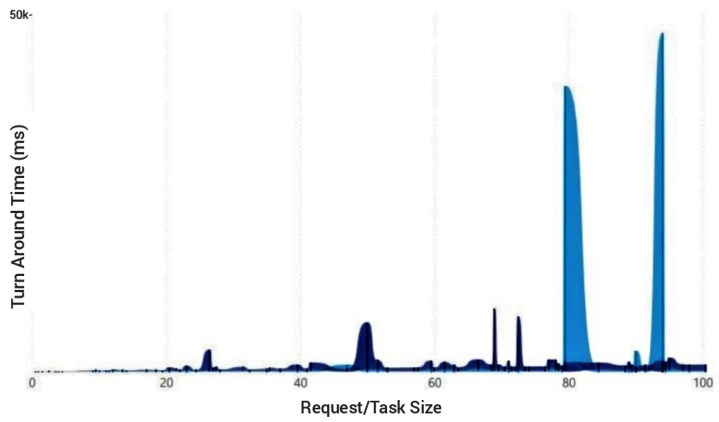
Cumulative turnaround time of C-LBCA vs PSOR2B.

The results presented in Figure 4, Figure 5 and Figure 6 display the individual strengths of each algorithm. For the sake of producing more optimal solutions and for comparisons with existing algorithms employed in the current state of the art, the secondary test scenario, depicted in Figure 7, Figure 8, Figure 9, Figure 10, Figure 11, Figure 12, Figure 13 and Figure 14, further exhibits the efficacy of the algorithms, not as individual algorithms this time but as a combined trio—PSOR2B in optimizing resource allocation in IoT.

The fourth (4th) test case therefore compares the trio combination (PSO + hybrid algorithm) against the PSO, as well as the trio combination against the hybrid algorithm. In both cases, the aim is to draw attention to the effectiveness of the proposed PSOR2B model, emphasising how it produces more optimal results in terms of the *TAT*. An improved *TAT* suggests improved power rates/battery lifespan and performance. Figure 7 and Figure 8 show how the proposed PSOR2B model is more efficient than the resource optimisation by the PSO methodology. The figures further show how the proposed model’s approach generates an efficient TAT better than the PSO’s approach, ranging between two hundred to three thousand, eight hundred milliseconds (200–3800 ms). Although the use of PSO on its own produces optimised results, a combination of PSO and the hybrid algorithm produces even better results. The few sudden hikes in the hybrid and PSO trio may be incidental to not finding the best fit at that given instance. Figure 9 and Figure 10 depicts how incorporating the PSO clustering algorithm to the hybrid RR-RB algorithm produces an even more optimal solution. The use of the hybrid algorithm produces a relatively good *TAT*; however, when PSO is incorporated, the *TAT* is seen to further reduce with the *TAT* closer to zero (0). This again shows the importance of the use of appropriate clustering techniques as an add-on and not stand-alone. It suffices to say therefore that the generated results depicted in Figure 9 and Figure 10 affirm the importance of the proposed PSOR2B. The PSOR2B algorithm harness the strengths of all three merged algorithms to produce a holistic algorithm that further optimizes IoT.

Figure 11 and Figure 12 give a summary of all outputs by giving comparisons of all three algorithms concerned. Given the establishment from preliminary test scenario three, as depicted in Figure 6, of the hybrid’s superiority over the PSO, the comparisons in Figure 11 and Figure 12 compare random allocation to hybrid and PSOR2B (hybrid algorithm + PSO) algorithms, giving at a glance the efficacy of PSOR2B. From previous test scenarios, it has been shown that the hybrid algorithm stands to produce more efficient outputs when compared to the current state of the art–random allocation—and the hybrid and PSO combination (PSOR2B) form an even more efficient technique for resource optimisation in IoT. Figure 11 and Figure 12 give a summary of this efficiency by comparing the three approaches of resource allocation: random, hybrid, and hybrid + PSO. The hybrid algorithm, which allocates resources based on request size whilst considering the resource capacity evidently shown in the figures, produces a more efficient TAT than the random allocation approach. Clustering by means of the addition of PSO focuses on dividing edge nodes into clusters consequently creating CHs; by creating clusters, the latency is hypothetically reduced by a factor of two. The effect of PSO is also seen in the figures as a combination of the hybrid and PSO algorithms generate an even more optimal result, with the TAT more than halved. In these depictions, the PSOR2B’s performance is consistently better than the random allocation and the previous improved hybrid algorithm. This affirms the importance of clustering algorithms in resource optimisation of IoT. It can therefore be inferred that the proposed topology further reduces the latency and enhances resource optimisation, as opposed to the random allocation where nodes have multiple connections. The proposed model limits the number of connections to servers via the clustering technique (PSO) to generate CHs. The CHs are key to the functionality of the hybrid algorithm as they aid in controlling the scheduling and allocation processes. The hybrid works on the foundation laid by the PSO as it leverages on the CH created by the PSO clustering algorithm to conduct the load balancing, thus further optimizing resource allocation in IoT.

Figure 13 and Figure 14 give the cumulative TAT for processing of one hundred requests; thus, the TAT runs into thousands of milliseconds contrary to the TAT depicted in previous test case scenarios. By developing the PSOR2B model, which focuses on the overall system performance, measured by TAT (turnaround time), energy dissipation is lowered. The incorporation of the EC paradigm in the model implies less transfer of data, and with less data transfer and the necessity for the processing of tasks, not only is energy dissipation controlled, the speed at which tasks are processed is improved, consequently improving QoS and bettering users’ QoE. The fifth and final test case scenario presents comparisons between the PSOR2B, LEACH, and C-LBCA. In Figure 13, the cumulative TAT for one hundred requests is portrayed. As can be seen, LEACH is the weakest of the three with a cumulative TAT of above two hundred thousand milliseconds, followed by the TAT for the C-LBCA of around fifteen thousand milliseconds. The PSOR2B, the strongest of the three, produces a cumulative output of ten thousand milliseconds. Based on these outputs, it can be inferred that CH rotation as incorporated in the LEACH algorithm is not a necessary focal point as other factors can be controlled to bring about a better TAT, and that improving the WSN lifespan, the focal point of the C-LBCA algorithm, has a gross effect on the TAT/overall system’s performance, considering the substantial improvements when likened to LEACH. The LEACH and C-LBCA have previously shown improvements in terms of energy dissipation and device lifespan; thus, PSOR2B’s superiority as depicted in Figure 13 indirectly shows its improvements on energy dissipation and device lifespan as these factors directly influence the overall system performance. The improvements seen in the PSOR2B over the C-LBCA can be credited to its ability to control the WSN performance via the clustering technique in addition to the RR-RB algorithm; aside from that, the implementation of the EC paradigm further improves systems processing prowess and security feature. Figure 14 gives a close-up comparison of the better performing C-LBCA in light blue with the proposed PSOR2B in dark blue. On a closer look, it can be drawn that the C-LBCA, which is a centralised approach, works more efficiently for smaller request sizes. PSOR2B, on the other hand, has a consistently low output for all request sizes and provides more efficient results for larger request sizes with fewer erratic tendencies. With the increasing popularity of IoT, the number of nodes and the request size are bound to increase exponentially; therefore, the centralised C-LBCA model, when compared to the decentralised PSOR2B, loses its relevance in terms of overall system performance. Since energy consumption amongst other factors directly affect a system’s performance, it suffices to say that that the larger the number of requests and varying request sizes, the less the effectiveness of the C-LBCA in comparison to the PSOR2B.

### 4.3. Limitations and Future Work

The PSOR2B, a merger of the EC paradigm, clustering techniques and the RR-RB hybrid algorithm work together for efficient, flexible, and scalable resource optimisation in IoT. PSOR2B’s employment of the incorporated concepts ensures its adaptability and consequent efficiency in varying network conditions. The PSOR2B’s capacity as illustrated in the generated results, taking into consideration the devices’ varying storage, processing, and computation capacity, explicitly portrays its efficiency as a solution for the MORAP IoT problem. Despite these positives, security provided by the incorporated EC paradigm can be further improved. Future work can also explore the implementation of the PSOR2B model in real-life scenario. Although the results generated suggest PSOR2B’s scalability, especially in the number of requests it can handle, a further implementation with a focus on PSOR2B’s performance in large-scale IoT can be further explored.

## 5. Conclusions

In this paper, an efficient resource optimisation algorithm for IoT labelled PSOR2B was developed. The algorithm utilizes aspects of the EC paradigm, the PSO clustering technique, round robin, and resource-based algorithms. Incorporating the advantages of the trio, the PSOR2B solves the resource optimisation problem in IoT, ensuring that equal importance is placed on resource allocation and resource scheduling. Given that IoT tends to grow in complexity with an increase in size, the designed and developed PSOR2B model abates the complexity problem by tackling the allocation and scheduling problems, consequently providing solutions for scalability and latency issues that directly influence QoS and users’ QoE.

The experimental results show the proposed PSOR2B’s efficient solutions for the resource optimisation problem in comparison to the existing ant colony and the LEACH and C-LBCA algorithms. Once the right task is assigned to the right resource, an optimised turnaround time (TAT) is inevitable. An efficient execution time implies an excellent TAT, and an improved TAT indicates less energy dissipation at nodal levels. Less energy dissipation promotes a longer lifetime for all edge nodes and subsequently the entire system. The results indicating the efficiency of the PSOR2B imply that QoS and consequently users’ QoE is assured. The promising results inevitably show the effectiveness of using clustering algorithms on existing algorithms to further resource optimisation in IoT. It suffices to say that although certain parameters are controlled, the random nature of data transfer and communication between nodes are not controlled, therefore reflecting real-life situations. The proposed model can therefore be replicated easily into real life situations with little or no adjustments. The results generated have been able to shift the focus to factors worth more consideration, such as taking into account and placing equal importance on resource scheduling and resource allocation; the request size and server/resource size; how efficiently these resources are used; and how situations such as bottleneck can be avoided.

Future work can further improve this model by making it secure, thus tackling the problems associated with IoT’s privacy and security, consequential of decentralising the IoT’s topology. The PSOR2B model can further be deployed into real life scenarios.

## Figures and Tables

**Table 1:** Simulation parameters.

Parameter	Value
Edge nodes	1–100
Servers	5
Number of simulation iterations	20
Monitoring area for each cluster	20 m × 20 m (400 m${}^{2})$
Packet size	≥168 bits
Message size	≥4608 bits

## References

[1] Lanlan K., Ruey-Shun C., Wenliang C., Yeh-Cheng C., Yu-Xi H. (2020). Mechanism analysis of non-inertial particle swarm optimisation for Internet of Things in edge computing. Eng. Appl. Artif. Intell..

[2] Qui T., Bolun L., Xiaobo Z., Houbing S., Ivan L., Jaime L. (2020). A Novel Shortcut Addition Algorithm with Particle Swarm for Multisink Internet of Things. IEEE Trans. Ind. Inform..

[3] Lakhan A., Mohammed M.A., Abdulkareem K.H., Jaber M.M., Nedoma J., Martinek R., Zmij P. (2022). Delay Optimal Schemes for Internet of Things Applications in Heterogeneous Edge Cloud Computing Networks. Sensors.

[4] Shafique K., Khawaja B.A., Sabir F., Qazi S., Mustaqim M. (2020). Internet of Things (IoT) for Next-Generation Smart Systems: A Review of Current Challenges, Future Trends and Prospects for Emerging 5G-IoT Scenarios. IEEE Access.

[5] Moghaddam S.M., O’Sullivan M., Unsworth C.P., Piraghaj S.F., Walker C. (2021). Metrics for improving the management of Cloud environments — Load balancing using measures of Quality of Service, Service Level Agreement Violations and energy consumption. Future Gener. Comput. Syst..

[6] Srinadh V., Rao P.V.N. Implementation of Dynamic Resource Allocation using Adaptive Fuzzy Multi-Objective Genetic Algorithm for IoT based Cloud System. Proceedings of the 2022 4th International Conference on Smart Systems and Inventive Technology (ICSSIT).

[7] Peng-Yeng Y., Jing-Yu W. (2008). Optimal multiple-objective resource allocation using hybrid particle swarm optimisation and adaptive resource bounds technique. J. Comput. Appl. Math..

[8] Nguyen T.N., Ambarani K.J., Thai M.T. (2022). Optimizing Resource Allocation and VNF Embedding in RAN Slicing. arXiv.

[9] Jooya A.Z., Baniasadi A., Analoui M. (2011). History-aware, resource-based dynamic scheduling for heterogeneous multi-core processors. Iet Comput. Digit. Tech..

[10] Gong Y.J., Zhang J., Chung H.S.H., Chen W.N., Zhan Z.H., Li Y., Shi Y.H. (2012). An Efficient Resource Allocation Scheme Using Particle Swarm Optimisation. IEEE Trans. Evol. Comput..

[11] Yan Y., Wang H., Tao Q., Fan W., Lin T., Xiao Y. (2020). Noncyclic Scheduling of Multi-Cluster Tools With Residency Constraints Based on Pareto Optimisation. IEEE Trans. Semicond. Manuf..

[12] Prasanth A., Jayachitra S. (2020). A novel multi-objective optimisation strategy for enhancing quality of service in IoT-enabled WSN applications. Peer-Peer Netw. Appl..

[13] Xueqiang Y., Shining L., Yun L. (2019). A Novel Hierarchical Data Aggregation with Particle Swarm Optimisation for Internet of Things. Mob. Netw. Appl..

[14] Al-Janabi T.A., Al-Raweshidy H.S. Optimised clustering algorithm-based centralised architecture for load balancing in IoT network. Proceedings of the 2017 International Symposium on Wireless Communication Systems (ISWCS).

[15] Lina X., O’ Hare G.M.P., Rem C. (2017). A Smart and Balanced Energy-Efficient Multihop Clustering Algorithm (Smart-BEEM) for MIMO IoT Systems in Future Networks. Sensors.

[16] Deng S., Xiang Z., Zhao P., Taheri J., Gao H., Yin J., Zomaya A.Y. (2020). Dynamical Resource Allocation in Edge for Trustable Internet-of-Things Systems: A Reinforcement Learning Method. IEEE Trans. Ind. Informatics.

[17] Wang N., Wang J.S., Zhu L.F., Wang H.Y., Wang G. (2021). A Novel Dynamic Clustering Method by Integrating Marine Predators Algorithm and Particle Swarm Optimisation Algorithm. IEEE Access.

[18] Pratyay K., Prasanta K.J. (2014). A novel differential evolution based clustering algorithm for wireless sensor networks. Appl. Soft Comput..

[19] Le H., Hoang D., Poliah R., Takizawa M., Barolli L., Enokido T. (2008). S-Web: An Efficient and Self-organizing Wireless Sensor Networsk Model. Lecture Notes in Computer Science, Proceedings of the Network-Based Information Systems, Turin, Italy, 1–5 September 2008.

[20] Pal R., Sharma A.K. FSEP-E: Enhanced stable election protocol based on fuzzy Logic for cluster head selection in WSNs. Proceedings of the 2013 Sixth International Conference on Contemporary Computing (IC3).

[21] Bi F., Zhou H., Zhu M., Wang W. (2022). Economic benefit evaluation of water resources allocation in transboundary basins based on particle swarm optimisation algorithm and cooperative game model—A case study of Lancang-Mekong River Basin. PLoS ONE.

[22] Wan X., Wang Z., Wu M., Liu X. (2019). *H*_∞_ State Estimation for Discrete-Time Nonlinear Singularly Perturbed Complex Networks Under the Round-Robin Protocol. IEEE Trans. Neural Networks Learn. Syst..

[23] Jun S., Zidong W., Yugang N. (2018). Static output-feedback sliding mode control under round-robin protocol. Int. J. Robust Nonlinear Control..

[24] Hameed Abdulkareem K., Awad Mutlag A., Musa Dinar A., Frnda J., Abed Mohammed M., Hasan Zayr F., Lakhan A., Kadry S., Ali Khattak H., Nedoma J. (2022). Smart Healthcare System for Severity Prediction and Critical Tasks Management of COVID-19 Patients in IoT-Fog Computing Environments. Comput. Intell. Neurosci..

[25] Lakhan A., Mohammed M.A., Elhoseny M., Alshehri M.D., Abdulkareem K.H. (2022). Blockchain multi-objective optimisation approach-enabled secure and cost-efficient scheduling for the Internet of Medical Things (IoMT) in fog-cloud system. Soft Computing.

[26] Alatoun K., Matrouk K., Mohammed M.A., Nedoma J., Martinek R., Zmij P. (2022). A Novel Low-Latency and Energy-Efficient Task Scheduling Framework for Internet of Medical Things in an Edge Fog Cloud System. Sensors.

[27] Lakhan A., Mohammed M.A., Garcia-Zapirain B., Nedoma J., Martinek R., Tiwari P., Kumar N. (2022). Fully Homomorphic Enabled Secure Task Offloading and Scheduling System for Transport Applications. IEEE Trans. Veh. Technol..

[28] Qiao Z. (2022). Research on Optimisation Algorithm of Cloud Computing Resource Allocation for Internet of Things Engineering Based on Improved Ant Colony Algorithm. Math. Probl. Eng..

